# Role of surgical modality and timing of surgery as clinical outcome predictors following acute subdural hematoma evacuation

**DOI:** 10.12669/pjms.36.3.1771

**Published:** 2020

**Authors:** Imran Altaf, Shahzad Shams, Anjum Habib Vohra

**Affiliations:** 1 Dr. Imran Altaf, MS. Department of Neurosurgery, Khawja Muhammad Safdar Medical College, Sialkot, Pakistan; 2 Dr. Shahzad Shams, FRCS, FCPS. Department of Neurosurgery, King Edward Medical University, **Mayo Hospital**, Lahore, Pakistan; 3 Dr. Anjum Habib Vohra, FRCS. Department of Neurosurgery, Post Graduate Medical Institute, Lahore General Hospital, Lahore, Pakistan

**Keywords:** Acute subdural hematoma, Craniotomy, Decompressive craniectomy, Timing of surgery

## Abstract

**Background & Objective:**

A Craniotomy (CO) or decompressive craniectomy (DC) are the two main surgical procedures employed for evacuation of acute traumatic subdural hematoma (ASDH). However, the optimal surgical procedure remains controversial. The beneficial effect of early surgical evacuation of acute subdural hematoma in improving outcome also remains unclear. Our objective was to study the role of these two parameters in determining the outcome in patients undergoing surgical evacuation of acute traumatic subdural hematoma.

**Methods:**

A retrospective analysis of 58 patients presenting with acute traumatic subdural hematoma and with presenting Glasgow Coma Scale (GCS) ≤ 8 that had been operated in Lahore General Hospital between June 2014 and July 2015 was performed. The demographic data, preoperative GCS, type of surgical procedure performed and timing of surgery were analysed.

**Results:**

Forty (69%) patients underwent CO, and eighteen (31%) patients underwent DC. The CO and DC groups showed no difference in the demographic data and preoperative GCS. Six patients survived in the craniotomy group, while none survived in the decompressive craniectomy group (p=0.083). The relationship of timing of surgery with survival in the craniotomy group was found not to be clinically significant (p=0.87).

**Conclusion:**

In this study craniotomy was associated with a better outcome as compared to decompressive craniectomy, however, the difference did not reach statistical significance. Early surgery was also found not to be associated with an improved outcome.

## INTRODUCTION

Acute subdural hematoma is considered to be the most lethal traumatic brain injury.^1^ Mortality is high and reported mortality ranges from 40-90%.^2^-^4^ A craniotomy and decompressive craniectomy are the two main surgical options employed for evacuation of acute traumatic subdural hematoma, but studies comparing their outcome have shown inconsistent results.^3^,^5^,^6^ The optimal surgical procedure in patients presenting with traumatic acute subdural hematoma still to date remains controversial.^6^-^8^ The role of timing of surgery as a predictor of outcome also remains unclear, and whether early surgery improves outcome still remains controversial.^1^,^9^ The present study was designed to assess the role of these two parameters as predictors of clinical outcome in patients presenting with acute traumatic subdural hematoma.

## METHODS

In this retrospective study, medical records of 58 patients with presenting GCS ≤ 8 who had undergone surgical evacuation of acute traumatic SDH from June 2014 to July 2015 at the Department of Neurosurgery, Lahore General Hospital were analyzed. Adult patients of both sexes that had been operated for acute subdural hematoma were included in the study. Acute subdural hematoma was diagnosed on CT (computed tomography) brain in all the patients. CT brain on admission was assessed for hematoma thickness and amount of midline shift. Surgical intervention either through a craniotomy or a craniectomy had been carried out in patients having a hematoma thickness of more than 10 mm on CT and a midline shift of more than 5 mm. Data was retrieved from the medical records of patients included in the study and the parameters of age, sex, preoperative GCS, surgical procedure performed and the timing of surgery were studied. Patients having concomitant intracranial pathology like a large contusion, traumatic ICH (intracerebral hematoma) or extradural hematoma were excluded. Also patients presenting with spontaneous ASDH were excluded from the study. For the surviving patients the outcome was categorized according to the Glasgow Outcome Scale (GOS) with the Outcome being classified as “favorable” if the GOS score was 4 or 5, and as “unfavorable” if GOS score was 3 or less.

Patients undergoing craniotomy were operated through a standard question mark incision followed by a frontotemporoparietal craniotomy. A decompressive craniectomy was performed either through a question mark incision and removal of the bone flap, or a linear incision over the temporal region with a large craniectomy. Dura was opened and the hematoma evacuated in all the cases.

### Statistical Analysis

The craniotomy and decompressive craniectomy group were compared for age and preoperative GCS using an independent t-test, and compared for sex using a chi-square test. A chi-square test was also used to assess the relationship of survival with the type of surgical procedure performed and the timing of surgery. For all analysis, a p-value of <0.05 was considered statistically significant.

### Ethics committee approval

This retrospective study was approved by ethics committee on November 11, 2019, Research No. 0097-19.

## RESULTS

Fifty-eight patients presenting with traumatic acute subdural hematoma and meeting the inclusion criteria were included. 40 (69%) patients underwent CO, and 18 (31%) patients underwent DC. Mean age of CO group was 42.8(SD 18.4) years, and of DC group was 47.8(SD 20.8) years. Overall there were 44 males and 14 females. The mean preoperative GCS of patients in the craniotomy group was 5.7(SD 1.32), and in the decompressive craniectomy was 5(SD 1.45). A comparison of age, gender and preoperative GCS between the craniotomy and decompressive craniectomy group did not show a significant difference between the two groups (Table-I).

**Table-I:** Comparison of patient demographics and preoperative GCS between the craniotomy and the craniectomy groups.

	Craniotomy	Decompressive craniectomy	P - value
*Gender*			
Male	29	15	0.3724
Female	11	3	
*Age*			
Mean ± SD (years)	42.78 ± 18.39	47.83 ± 18.48	0.3374
*Pre-operative GCS*			
Mean ± SD	5.7±1.32	5 ±1.46	0.07624

None of the eighteen patients in the decompressive craniectomy group survived (100% mortality). Six of the forty patients in the craniotomy survived (85% mortality), with four having a functionally good outcome. Overall mortality was 89.6%. The difference between the two groups was, however, not significant (p=0.083) as shown in Table-II.

**Table-II:** Comparison of type of surgical procedure performed between the survivors and non-survivors.

Procedure	Survivor	Non survivor	P - value
Craniotomy	6	34	0.08269
Decompressive craniectomy	0	18	
Total	6	52	

The timing of surgery as a predictor of survival was analyzed in the craniotomy group. The relationship with survival was found not to be statistically significant (p=0.87) as shown in Table-III.

**Table-III:** Relationship of timing of surgery with survival.

Timing of surgery (Hours)	survivor	Non survivor	P - value
≤ 4	2	8	0.8693
4 - 10	2	14	
≥ 10	2	12	

## DISCUSSION

Acute subdural hematoma is present in about one third of patients presenting with severe traumatic brain injuries.^10^,^11^ The mortality is high despite advances in emergency medical care and surgical techniques, and ASDH remains one of the most lethal intracranial injuries.^10^ Indications for surgical evacuation of ASDH include a thickness greater than 10 mm, or midline shift greater than 5mm on CT brain.^12^,^13^ In principle, the purpose of surgery is to decrease intracranial hypertension to prevent brain from secondary injury.^9^ Various surgical modalities such as simple burr hole trephination, CO and DC are used for evacuation of ASDH,^9^,^10^,^14^ but the superiority of either procedure has not yet been established.^9^,^10^

The rationale behind performing DC is that DC gives flexible ICP (intracranial pressure) control and provides extra space for edematous brain tissue.^8^,^10^ However performing CO or DC for ASDH remains controversial as studies comparing them have shown conflicting results.^3^,^5^,^6^,^8^,^15^ Generally it is held that the results of CO are superior to that of DC.^6^,^7^,^10^,^16^,^17^ These findings, however, get complicated by the fact that patients in whom DC was carried out were in poor clinical status, and thus had an intrinsic layout for a poor outcome.^6^,^10^,^16^,^17^,^18^ Also in many of these studies although the outcome in the CO group was better, yet the comparison with the DC group showed that in some cases the difference was significant,^5^,^6^,^10^,^17^ while in others the difference was not clinically significant.^16^,^18^ This has led to a wide variation in the clinical practice of neurosurgeons around the world with some neurosurgical institutes using decompressive craniectomy, whilst others using cranioplastic craniotomies while dealing with acute subdural hematoma.^7^,^9^,^19^ In our study for the sake of uniformity we included only patients in whom the presenting GCS was ≤ 8. We found that all the six survivors were in the CO group with four having a good outcome. None of the patients in the DC group survived. The difference in outcome, however, did not reach clinical significance (p=0.083). Our findings are thus consistent with the findings of Li LM et al.^16^ and Woertgen C et al.^18^ that also found that although the mortality was higher in the craniectomy group as compared to the craniotomy group yet the difference did not reach clinical significance.

The timing of surgery as a predictor of outcome after surgical evacuation of ASDH remains a controversial topic.^1^,^9^,^12^ Seelig et al.^20^ in their land mark study found that patients operated within 4 hours after trauma had a survival rate of 90%, as compared to a survival rate of only 30% for patients operated after four hours. But since then cohort studies from Canada, Poland, USA and UK found that timing of surgery was not predictive of outcome.^1^ Infect, paradoxically, Walcott et al^21^ found that increased time from trauma to surgery significantly reduced mortality in patients undergoing operative treatment of traumatic ASDH. We also found that timing of surgery did not have a relationship with outcome (p=0. 87) after surgical evacuation of ASDH.

### Limitations of the study

This is a small size retrospective, non-randomized, single center study and thus potentially subject to diverse biases and variations. We believe that further investigation with a larger sample size, quantitative controlled prospective study is required to clarify the role of these two parameters as predictors of clinical outcome following acute subdural hematoma evacuation.

## CONCLUSION

In this study craniotomy was associated with a better outcome as compared to craniectomy, however, the difference did not reach statistical significance. Early surgery was also found not to be associated with an improved outcome.

### Authors’ Contribution:

**IA** conceived, designed, did data collection & statistical analysis & manuscript writing & editing of manuscript & is responsible for integrity of research.

**SS & AHV** did review and final approval of manuscript.
